# Aligning Developmental and Processing Accounts of Implicit and Statistical Learning

**DOI:** 10.1111/tops.12396

**Published:** 2018-11-09

**Authors:** Michelle S. Peter, Caroline F. Rowland

**Affiliations:** ^1^ Department of Psychological Sciences ESRC LuCiD Centre University of Liverpool; ^2^ Language Development Department Max Planck Institute for Psycholinguistics

**Keywords:** Syntax acquisition, Error‐based learning, Implicit learning, Statistical learning, Structural priming, Dual‐path model, Prediction

## Abstract

A long‐standing question in child language research concerns how children achieve mature syntactic knowledge in the face of a complex linguistic environment. A widely accepted view is that this process involves extracting distributional regularities from the environment in a manner that is incidental and happens, for the most part, without the learner's awareness. In this way, the debate speaks to two associated but separate literatures in language acquisition: statistical learning and implicit learning. Both fields have explored this issue in some depth but, at present, neither the results from the infant studies used by the statistical learning literature nor the artificial grammar learning tasks studies from the implicit learning literature can be used to fully explain how children's syntax becomes adult‐like. In this work, we consider an alternative explanation—that children use error‐based learning to become mature syntax users. We discuss this proposal in the light of the behavioral findings from structural priming studies and the computational findings from Chang, Dell, and Bock's (2006) dual‐path model, which incorporates properties from both statistical and implicit learning, and offers an explanation for syntax learning and structural priming using a common error‐based learning mechanism. We then turn our attention to future directions for the field, here suggesting how structural priming might inform the statistical learning and implicit learning literature on the nature of the learning mechanism.

## Abstract 2

In this article, Peter and Rowland explore the role of implicit statistical learning in syntactic development. It is often accepted that the processes observed in classic implicit learning or statistical learning experiments play an important role in the acquisition of natural language syntax. As Peter and Rowland point out, however, the results from neither research strand can be used to fully explain how children's syntax becomes adult‐like. They propose to address this shortcoming by using the structural priming paradigm.

## 1 Introduction

To form grammatical utterances, children must assign words to the different syntactic categories required by their language and combine these categories according to particular syntactic rules to convey meaning. What is remarkable about this process is that children do this with no formal teaching about how these categories operate. Thus, to learn syntax, children must be able to keep track of a range of abstract, complex, and often seemingly arbitrary syntactic patterns in their input. This is no mean feat. For example, among multiple other rules, they must learn the grammatical marking of semantic roles such as agent and patient, as well as how to map these semantic roles onto syntactic positions (e.g., subject and object). They also need to learn that, in some languages, altering word order can have semantic consequences (e.g., *The girl pushed the boy* means something different from *The boy pushed the girl*), and that not all verbs can be used in the same way (e.g., *fight* can be used both intransitively [*The boy fought*] and transitively [*The boy fought his opponent*]*,* but *swim* cannot [*The boy swam*] vs. [*The boy swam his opponent]). How then do children manage to develop adult‐like syntactic knowledge without receiving any explicit explanation of what is acceptable and what is not?

One idea is that children have innate linguistic knowledge which guides their interpretation of the input, allowing them to form abstract syntactic representations from early on (early abstraction theories; e.g., Chomsky, 1965; Gertner, Fisher, & Eisengart, 2006; Hirsh‐Pasek & Golinkoff, 1996; Jin & Fisher, 2014; Naigles, 1990, 2002; Pinker, 1984; Valian, 1986; Yuan, Fisher, & Snedeker, 2012). An alternative view is that, instead, syntactic representations are initially built around item‐specific schemas (e.g., knowledge of how the verb *go* behaves in sentences), but gradually become abstract through a process of learning and generalization (lexical constructivist theories; e.g., Abbot‐Smith, Lieven, & Tomasello, 2001; Bannard & Matthews, 2011; Goldberg, 1999; Lieven, Pine, & Baldwin, 1997; Matthews, Lieven, Theakston, & Tomasello, 2005; Olguin & Tomasello, 1993; Pine, Lieven, & Rowland, 1998; Rubino & Pine, 1998; Tomasello, 1992, 2008). The main points of contention between the theories focus on what kind of innate knowledge children bring to the learning task, and whether children achieve mature syntactic knowledge early, as a result of powerful innate syntactic knowledge, or late, in the absence of such knowledge. However, both theories agree that statistical learning mechanisms must play a part, and that a large part of syntax acquisition involves extracting distributional regularities from the environment—a skill not limited to language learning (Cartwright & Brent, 1997; Gerken, Wilson, & Lewis, 2005; Redington, Chater, & Finch, 1998). For syntax to be acquired in this way, the learning mechanisms involved must, at the very least, be sensitive to statistical cues present in the input. In addition, since the rules about the co‐occurrence of words are never formally explained to children, this learning must be incidental. Thus, research on syntax acquisition can inform, and be informed by, two associated but separate literatures: statistical learning and implicit learning.

## 2 Statistical learning and implicit learning: What can they tell us about syntax acquisition?

The brain is able to detect, keep track of, and learn from the vast number of regularities in a complex, sensory environment. This type of learning—*statistical learning*—is incidental (occurring even when the learner is not intending to learn), is not limited to one aspect of cognition, and is not even a uniquely human ability. It has been observed in the visual processing of shapes (e.g., Fiser & Aslin, 2002; Kirkham, Slemmer, & Johnson, 2002) and in the auditory processing of tone sequences (e.g., Creel, Newport, & Aslin, 2004; Saffran, Johnson, Aslin, & Newport, 1999), and it occurs in non‐human primates such as apes (e.g., Rakoczy et al., 2014) and baboons (e.g., Goujon & Fagot, 2013). Statistical learning has also has been shown to play a role in some linguistic processes, providing researchers of language acquisition with an explanation for the rapidity with which young children demonstrate acquisition of complex linguistic knowledge despite no explicit instruction. For instance, there is evidence to suggest that statistical learning is involved in children's phonetic learning (e.g., Maye, Weiss, & Aslin, 2008) and in their ability to segment words from speech (e.g., Mintz, 1996). Probably the most influential evidence for the latter comes from work by Saffran, Aslin, and Newport (1996), which revealed that children as young as 8 months old are sensitive to the conditional probabilities in the environment such that they are able to pick up on the sequential statistics of an artificial language. In this seminal study, 8‐month‐olds used distributional cues such as the transitional probabilities between syllables within and between words (i.e., the probability that syllable A will be followed by syllable B) to learn word boundaries from as little as 2 min of exposure to pseudospeech. Since then, a number of other studies have demonstrated similar findings (e.g., Aslin, Saffran, & Newport, 1998; Johnson & Jusczyk, 2001; Thiessen & Saffran, 2003), providing compelling evidence that very young children employ powerful statistical learning abilities during some aspects of the early stages of language acquisition.

As well as using statistical cues as a source of information about word boundaries, studies have suggested that infants are also able to exploit the regularities in the environment to extract rudimentary syntax‐like rules (e.g., Saffran & Wilson, 2003). In a task by Gomez and Gerken (1999), 12‐month‐olds were exposed to a subset of strings (e.g., VOT PEL JIC and PEL TAM PEL JIC) from one of two artificial grammars. They then listened to a grammar comprising strings that either agreed with or violated the underlying structure of the training grammar. Children showed a preference for new strings that shared the structure of the training grammar over new strings generated by a different grammar. Findings like this, in which young children demonstrate the ability to quickly generalize their knowledge to discriminate new grammatical from ungrammatical strings, has added weight to the proposal that the abstraction of syntax‐like regularities from distributional patterns in the input is underpinned by a mechanism that uses statistical learning.

An important feature of this type of learning is that it can occur without an individual's conscious awareness; participants tend not to demonstrate knowledge about their knowledge (e.g., Kim, Seitz, Feenstra, & Shams, 2009; Turk‐Browne, Junge, & Scholl, 2005). Because of this, statistical learning has been described as occurring implicitly (e.g., Goujon, Didierjean, & Thorpe, 2015)—a characteristic that suggests a degree of alignment with the field of implicit learning.

Coined by Reber (1967), *implicit learning* describes a process that results in the acquisition of abstract knowledge and happens in the absence of knowledge about how this learning has been achieved. Much of the evidence for implicit learning in language acquisition comes from artificial grammar learning tasks (AGL) in which participants are told to remember a number of short letter‐strings that appear to be arbitrary but are actually constructed according to a finite‐state grammar (a finite set of linear rules by which an infinite number of sentences can be produced). Participants are trained on a subset of letter‐strings, before having to generalize this knowledge to new strings. Remarkably, adults are sensitive to the grammatical nature of these types of stimuli even if exposure to the language is brief (e.g., Perruchet & Pacteau, 1990). Furthermore, when asked explicitly, they are unable to reveal much information about the rules by which they have generated these new strings (Reber, 1967; Reber & Allen, 1978). Thus, it seems that adults are able to extract syntax‐like rules from linear distributional information, with the literature on AGL converging on the view that the mechanism involved in the formation of abstract syntax uses implicit learning.

It is clear that there are aspects on which both the statistical learning and implicit learning communities are aligned. One striking similarity between the two literatures concerns the ability of humans to become sensitive to regularities in the environment and to use this information to make predictions and decisions about future experiences. Another concerns the learner's lack of awareness: The acquisition of knowledge without intending to learn, and without knowledge of the process, is a hallmark of both of these types of learning (though see Batterink, Reber, Neville, & Paller, 2015; Bertels, Franco, & Destrebecqz, 2012; who propose that statistical learning is not an exclusively unconscious process and might be accompanied by explicit knowledge). Both fields of research also use artificial languages to assess the nature of the learning mechanism. It is unsurprising, then, that the terms are sometimes used interchangeably and have even been combined (e.g., *implicit statistical learning*; Christiansen, 2018; Conway & Christiansen, 2006; Goujon et al., 2015).

Despite this overlap, however, there are important distinctions between the two fields. For instance, the implicit learning literature has concentrated on whether the mechanism can learn simple syntactic structures and whether this knowledge is acquired consciously, whereas statistical learning research has focused mainly on how the mechanism uses the probabilities between sequences to isolate words from speech (though see Frost & Monaghan, 2016). Consequently, a further difference concerns how both types of learning are operationalized in an experimental setting: Implicit learning has used serial reaction‐time tasks (e.g., Nissen & Bullemer, 1987) and AGL tasks (e.g., Reber, 1967), while statistical learning has typically used word segmentation tasks like those mentioned above (e.g., Aslin et al., 1998).

Even though, as we have mentioned, each strand approaches the issue from a different perspective, many researchers from these fields share the goal of wanting to better understand the process by which we acquire syntactic knowledge in the face of a complex linguistic environment. Remarkable progress has been made in this regard, with a clear consensus that even infant learners possess the capabilities to exploit the predictability of the underlying structure of the input. However, neither the results from the infant studies used by the statistical learning literature nor the AGL studies used by the implicit learning literature can, at present, be used to fully explain how children's syntax becomes adult‐like. This is because the findings from AGL studies can explain how learners unconsciously track the regularities of a grammar's surface structure, but not necessarily how this ability can be used to build knowledge about a grammar with a complex, hierarchical structure. Relatedly, although the findings from the statistical learning field provide compelling evidence that infants can use statistics to learn simple syntactic regularities, many of the studies have used artificial languages, meaning that assumptions about grammar learning are made based on learning regularities from an artificial speech stream. While there are advantages to using artificial languages (e.g., their use allows for a higher level of control over the input), they are, unfortunately, unable to fully capture the intricacies and complexities of natural language. This makes it difficult to scale these findings up to syntax learning in the real world. Furthermore, the findings from neither field are able to explain how young children make use of semantic information in the input during the formation of abstract syntactic categories, which must be part of the solution, since learning a grammar is essentially a process of learning to use syntactic structure to express meaning.

One way of reconciling these issues is to use an alternative method called the structural priming paradigm which exploits the tendency for speakers to re‐use the syntactic structure of the sentences that they have recently encountered (e.g., Bock, 1986). Typically, priming tasks use verbs that can alternate between structures that are semantically similar but are syntactically different. For example, the dative verb *give* alternates between the double object dative (DOD; *Wendy gave Bob a dog*) and the prepositional object dative (PD; *Wendy gave a dog to Bob*). Participants are usually presented with a prime sentence using a particular structural form (e.g., DOD; *Wendy gave Bob a dog*), and then produce a new sentence (a target) describing a different event/scene (e.g., DOD; *the boy threw the girl a ball*/PD; *the boy threw a ball to the girl*). Evidence of structural priming is demonstrated if a participant's target sentence uses the same syntactic structure as the prime. Since there are no similarities in lexical content (i.e., the prime verb and target verb are different), repetition of the prime's structure indicates that participants are primed by the structure and not the semantics of the sentence. As such, structural priming effects are interpreted as evidence of abstract syntactic knowledge (e.g., Cleland & Pickering, 2006; Noppeney & Price, 2004).

For some time now, researchers have capitalized on this linguistic phenomenon since priming effects bring together issues relevant to and have direct implications for a number of disciplines. They have been informative for understanding the architecture of the adult lexicon, the nature of adult syntactic representations, and for learning how the adult processing system works (see Pickering & Garrod, 2004, Ferreira & Bock, 2006, who suggest that priming serves as an important function in improving communication between interlocutors). Structural priming has also been influential in shaping our understanding of what children's early syntactic knowledge is like and, more recently, to study the transition between the two: how children's syntactic knowledge develops to become adult‐like. To add to this, priming has also been used to explore the types of learning mechanisms that might be involved in this process (Peter, Chang, Pine, Blything, & Rowland, 2015). Thus, it is clear that not only does structural priming have much to contribute to both the adult and child language literature, but it too is important for the statistical learning and implicit learning community who share the goal of wanting to better understand how syntactic knowledge is built.

Relatedly, there have been a number of attempts to model syntax acquisition. However, these models fail to include psychological and computational features that McCauley and Christiansen (2014) argue are important for a plausible model of this process. For instance, the authors propose that a model of syntax acquisition should (a) process input on‐line in a word‐by‐word manner, as opposed to learning entire utterances; (b) learn by calculating statistics that are tied to backward transitional probabilities as opposed to using only simple distributional information; and (c) be trained using naturalistic linguistic input as opposed to input that is artificial or lacks the properties of real language. Thus, models that do not model development incrementally (e.g., Bannard, Lieven, & Tomasello, 2009), focus only on simple distributional information (e.g., Redington et al., 1998), or are not fed input with the structure of real natural languages (e.g., Howell & Becker, 2001, in which the model is trained on a 390‐word language comprising two‐ and three‐word sentences) are not fully able to capture the process by which children develop adult‐like syntactic knowledge.

Perhaps more important, none of them provides an explanation that scales up to adult language use, incorporating an implicit learning explanation of why we see structural priming effects in both children and adults. Conversely, a number of models have been developed to capture structural priming effects (e.g., Pickering & Branigan, 1998; Reitter, Keller, & Moore, 2011; Tooley & Traxler, 2010). Yet, to our knowledge, these models do not tell us how children acquire syntactic knowledge to become mature syntax users.

There is, however, a model of syntax acquisition that has addressed these issues. Chang et al.'s (2006) dual‐path model uses an error‐based learning mechanism that incorporates properties from both statistical and implicit learning (a feature that we will return to discuss in due course) to describe how sentences are processed and how syntactic knowledge is built.

Although the model is primarily one of syntax acquisition, it is also able to explain structural priming in terms of the same error‐based mechanism. What is more, the model includes a semantic network to account for the parallel acquisition of knowledge about thematic relations and to encode the system's intended message. Further still, the assumptions made by the model are based on the properties of natural and not artificial language.

In this way, the dual‐path model offers an explanation for both syntax learning and structural priming effects using a common mechanism, considers the role of semantics in the formation of abstract syntactic knowledge, and develops this knowledge by tracking statistical regularities in real speech as opposed to the surface structure of an artificial language.

As such, this model is the one on which we have chosen to concentrate the rest of our discussion. First, we review the behavioral evidence to consider how young children might use error‐based learning to become mature syntax users and, in this way, examine how psychologically plausible the dual‐path is as a model of syntactic development. We then turn our attention to future directions for the field where we suggest how structural priming can add to the debate in the statistical learning and implicit learning literature on the nature of the learning mechanism.

## 3 Error‐based implicit learning as a mechanism for syntax acquisition: How does the dual‐path model work?

The dual‐path model conceptualizes the development of syntax in terms of an error‐based implicit learning mechanism with a dual‐pathway architecture comprising a simple recurrent network (SRN) and a (hidden) meaning network. The meaning network contains the intended message of the sentence which may be conveyed by a number of structures (e.g., the act of object transfer might be expressed by either a DOD or a PD). Syntax learning occurs because the system uses statistics to exploit the regularities of the linguistic input. By keeping track of the frequency of co‐occurring items—a process that happens implicitly—the system is able to use this knowledge to generate a prediction about the next word in a sentence based on sequential restraints (the previous word) and information from the meaning network about the type of message that is being conveyed (the context). It then calculates the difference (error) between the predicted and the actual word and uses this prediction error to make gradual changes in the weights that support syntactic knowledge in the system. Increasing experience and continual feedback strengthen the model's predictive abilities so that, gradually, it makes more accurate predictions about the next word in a sentence. This type of supervised learning enables the model to develop abstract syntactic categories and, using meaning, to sequence these categories to generate sentences. Thus, the small weight changes in the model that are made during this process eventually allow it to converge on the representations that support adult‐like sentence production.

Importantly, the same error‐based implicit learning mechanism that acquires abstract syntax also produces structural priming effects. When the model is tested for priming by presenting the prime sentence with error‐based learning left ON, the prediction error for the prime is used to make changes to the weights in the network—some of which are made to the model's abstract structural representations. These weight changes influence the model's target utterance, increasing the use of the same structure and creating a structural priming effect. Thus, the model provides an account in which syntax acquisition and abstract priming are the result of a common error‐based learning mechanism. What's more, this type of mechanism is able to explain a number of different phenomena observed in structural priming tasks. An important question, however, is whether the effects simulated by the model are supported by empirical evidence. That is, while the dual‐path model as a model of syntax acquisition is theoretically feasible, is it psychologically plausible?

## 4 Can children really use error‐based learning to learn syntax? What's the evidence?

The dual‐path model makes a number of predictions about syntactic development that can be tested using structural priming. One is that, via a process of error‐based learning, children implicitly learn syntactic categories and how to combine them into syntactic structures from early in the acquisition process. On this account, children should show effects of abstract priming as soon as they have acquired abstract structures (from around the age equivalent of 3 years old in the model). This prediction is upheld by a number of studies in which children as young as 3 years old have demonstrated evidence of abstract structural priming with the dative both in language production (e.g., Peter et al., 2015) and in comprehension (e.g., Thothathiri & Snedeker, 2008). For instance, in Rowland, Chang, Ambridge, Pine, and Lieven's (2012) task, children (aged 3–6 years) and adults completed target fragments (e.g., *The boy sent* ___) designed to elicit a dative response after hearing an experimenter describe cartoon animations using either a DOD (e.g., *Wendy gave Bob a puppy*) or a PD (e.g., *Wendy gave a puppy to Bob*) prime sentence. Rowland et al. found significant structural priming across development in that both children and adults produced more DOD responses after a DOD prime than after a PD prime. There is also evidence that children are primed by transitive structures (e.g., Branigan & Messenger, 2016; Messenger, Branigan, McLean, & Sorace, 2012). For example, in an early study by Bencini and Valian (2008), children primed with passives were significantly more likely to produce passive target sentences compared to those primed with actives and those not primed at all. These findings shed light on the nature of early syntactic knowledge: Evidence of abstract structural priming suggests that children as young as 3 years old, like adults, have acquired abstract syntactic knowledge which they use to generalize across similarly structured sentences. Crucial to this discussion, though, is that the behavioral findings also offer insight into the potential mechanism involved in the acquisition of this knowledge. In the dual‐path model, priming effects occur because prediction error for the prime sentence results in small adjustments to abstract structural representations. This influences the structure choice of the target by slightly biasing it towards the structure of the prime. Thus, the demonstration of priming effects in the child studies can be used to support a model of syntax acquisition in which children use error‐based learning to make predictions about the language that they are experiencing.

The dual‐path model's implementation of syntax acquisition as slow, error‐based learning can also be used to explain how verb‐structure preferences (i.e., that certain verbs are more likely to occur in one syntactic structure than another) are acquired. These probabilistic verb‐structure preferences or verb biases are learned because incremental adjustments to the language system are made each time a verb is presented in a particular structure.

An inevitable by‐product of verb bias acquisition is prime surprisal—a phenomenon that affects performance in structural priming tasks. Prime surprisal is the result of a mismatch between the predicted next word (based on knowledge of verb biases) and the actual next word. For example, a prime sentence in which a DOD‐biased verb is presented in a PD structure (e.g., *The girl gave a book to the man)* is more surprising than a prime sentence in which both verb bias and verb structure are matched (e.g., DOD‐biased verb in a DOD structure; *The girl gave the man a book*). Because prime sentences with verb‐structure mismatches diverge from the system's expectation, they yield a greater amount of error. This leads to larger changes to connection weights in the underlying language network so that the structure of the target sentence is more likely to match that of the prime. Put simply, structural priming effects get stronger as the prime sentence becomes more surprising. In addition, because the same mechanism that learns abstract syntactic structure is also able to learn verb biases (Chang, Janciauskas, & Fitz, 2012), prime surprisal effects should be observable from the age at which abstract structural priming is demonstrated (i.e., 3 years old).

Consistent with this idea, a number of experimental findings have indicated that adults are indeed sensitive to verb‐structure mismatches of the type mentioned above. For example, Jaeger and Snider (2007) re‐analyzed the dative structures in a corpus of speech by Bresnan, Cueni, Nikitina, and Baayen (2007) and found that priming was stronger for PD primes if the verb in that prime was DOD‐biased. Jaeger and Snider (2013) also showed that adults were more likely to be primed when the co‐occurrence of the prime verb and prime structure was unexpected: Their corpus analysis study showed that adults were more strongly primed when DOD‐biased prime verbs were presented in a PD prime structure. Similar effects have also been found in language production in Dutch (Bernolet & Hartsuiker, 2010). This hypothesis has recently been tested in children, with findings indicating that, like adults, they are also sensitive to verb‐structure mismatches. Peter et al. (2015) manipulated prime surprisal by having verbs with biases that matched or mismatched the prime structure for both children (aged 3–6 years) and adults, and reported that children showed stronger priming effects when there was a mismatch between the prime verb's bias and the prime structure. The behavioral research, therefore, does seem to support the computational findings. As with abstract priming effects, prime surprisal effects are observable in children from as young as 3 years old, adding weight to the idea that both the acquisition of abstract syntax and the development of verb‐structure links occurs via a process of error‐based implicit learning.

The priming effects that have been discussed so far have all occurred in cases where the target sentence is immediately preceded by the prime. Structural priming, however, even occurs when there is intervening material between prime and target sentences. In other words, while these effects can be shortterm, they can also persist over time. Both immediate and long‐term priming can be explained in terms of a mechanism that uses error‐based implicit learning; this was tested when Chang et al. (2006) presented the dual‐path model with dative and transitive prime‐target sentences interspersed with intransitive fillers. Leaving the learning mechanism ON during processing of the prime led to the same type of changes in the system's internal abstract representations as when prime‐target pairs did not include fillers. As such, despite having to process as many as 10 filler sentences, the model still tended to use the prime's structure to describe the target message.

A number of behavioral studies seem to support this notion of longer term linguistic adaptation (e.g., Bock, Dell, Chang, & Onishi, 2007). For instance, Hartsuiker and Kolk (1998) manipulated the number of filler sentences between primes and targets and found that structural priming effects in adults were long‐lasting, and work by Bock and Griffin (2000) indicated that adults were primed even when there was intervening material (up to ten filler sentences) between primes and targets. Long‐term priming effects have also been demonstrated in comprehension; Tooley, Swaab, Boudewyn, Zirnstein and Traxler's (2014)'s study revealed that adults’ processing of target sentences was facilitated despite three fillers appearing between prime and target sentences. Similar effects have been demonstrated in studies with children (Huttenlocher, Vasilyeva, & Shimpi, 2004; Kidd, 2012; Savage, Lieven, Theakston, & Tomasello, 2003, 2006). Thus, the experimental findings seems to fit with Chang et al.'s (2006) proposal: Prediction error for the prime (as a consequence of error‐based implicit learning) leads to small, but long‐term adjustments to abstract structural representations that, in turn, influence the structure choice of the target. Because, on this view, these adjustments are long‐lasting, the description of the target remains biased towards the prime structure even when there is intervening material between prime‐target pairs.

On balance, Chang et al.'s (2006) dual‐path model is currently one of our most plausible models of syntax acquisition: It can, using error‐based implicit learning, explain how children acquire simple abstract syntactic representations, how they learn to link these representations to their knowledge about how verbs behave, and, as such, how this knowledge adapts in response to the input. It is also able to explain why structural priming effects happen and can account for a range of phenomena observed in these tasks.

We note, however, that the model is not without its problems. One issue is that error‐based implicit learning cannot account for the lexical boost in priming—an effect whereby structural priming is stronger when lexical items (verbs in particular) are shared across prime and target sentences (e.g., a prime‐target pair with *give‐give* will prime more strongly than *give‐send*; Hartsuiker, Bernolet, Schoonbaert, Speybroeck, & Vanderelst, 2008; Pickering & Branigan, 1998). While structural priming effects are successfully conceptualized in terms of error‐based implicit learning, lexical boost effects are too large to be a result of this type of mechanism (e.g., the adults in Rowland et al., 2012 and Peter et al., 2015 showed a 34% and 23% boost to the priming effect, respectively, when verbs were repeated across sentences). Large weight changes in a model of this kind are risky because they can result in the destruction of existing knowledge by recently experienced input (McCloskey & Cohen, 1989). In response to the lexical boost findings, Chang et al. (2006) have proposed that the lexical boost relies on a separate explicit memory mechanism which creates large, short‐term effects that do not persist long enough to make changes to the language network (see also Bock & Griffin, 2000; Chang et al., 2012; for similar arguments for a separate mechanism). On this view, the lexical boost might be expected to grow in line with the development of explicit memory or is, at least, disconnected from the structural priming effect so, though we might sometimes see large and sometimes small lexical boost effects, we will always see roughly the same sized (small) structural priming effect. Co‐opting an additional (explicit) memory mechanism to explain the lexical boost clearly makes the model less parsimonious than other models of priming. However, there is some evidence to support this dual‐mechanism hypothesis. For instance, studies have revealed that while structural priming effects are long‐lasting, the lexical boost is more short‐lived, comparable to the time‐course of explicit memory traces, which have been shown to dissipate quickly (e.g., Hartsuiker & Kolk, 1998; Konopka & Bock, 2005). In addition, recent work has directly assessed and found support for the model's developmental predictions regarding the boost (e.g., Branigan & McLean, 2016; Peter et al., 2015; Rowland et al., 2012), making the proposal that the boost is underpinned by a mechanism separate to the one from which structural priming effects arise a stronger possibility.

At this point, it is important to reiterate that other accounts of structural priming provide more parsimonious explanations of the lexical boost. Pickering and Branigan's (1998) model, which uses a mechanism akin to associative learning, is based on the lexicon (made up of lemma and combinatorial nodes) having an architecture in which residual activation of a syntactic structure promotes the selection of that same structure. Unlike the dual‐path model, the residual activation account can successfully explain structural priming and lexical boost effects using the same mechanism. The account, however, has its own problems. First, it is not clear how syntactic information within the lexicon is acquired and subsequently develops. Therefore, there is no developmental component; the strength of syntactic representations is the same in adults as in children, contrary to the findings of different patterns of priming across development (e.g., Peter et al., 2015; Rowland et al., 2012). Second, because the model does not keep track of, nor learn from, distributional regularities in the input, it would, presumably, predict that the same magnitude of activation is required across all verbs and structures, regardless of frequency in the input or whether or not verbs express a preference for one syntactic structure over another. This is problematic since prime surprisal effects have been demonstrated in both children and adults. Third, since the activation of nodes within the lexicon is short‐lived, it is not clear how the model can account for priming effects that persist over time (though see Pickering, Branigan, & McLean, 2002, for a counterargument).

Other accounts have also had more success than the dual‐path model at capturing a range of priming effects within one model (e.g., Tooley & Traxler, 2010; Reitter et al., 2011; Malhotra, 2009). These accounts, like the dual‐path model, explain priming in terms of implicit learning, but the process is operationalized differently because of the differing architecture of systems. For instance, Tooley and Traxler's (2010) account uses a mechanism that incorporates both implicit learning and increased activation. In this model, priming occurs because of both increased activation of the combinatorial nodes (which encode syntactic information), and changes in the strength between these nodes and lemma nodes which is caused by implicit learning. In comparison, the type of unsupervised learning in Malhotra's (2009) model produces memory traces (rather than error as a result of predictions as in Chang et al.'s model) which the system uses for processing. Different still, in Reitter et al.'s (2011) model, which comprises an ACT‐R cognitive architecture, priming is the result of base‐level and spreading activation from lexical to syntactic representations via associative links. Despite these differences, because these accounts comprise both a long‐term mechanism for adaptation and a short‐term mechanism to produce immediate effects, they are all able to explain a range of phenomena including structural priming, the lexical boost, and cumulative priming (whereby priming is larger after exposure to multiple primes of the same structure; Kaschak, 2007; Kaschak & Borreggine, 2008; Kaschak, Kutta, & Jones, 2011; Kaschak, Loney, & Borreggine, 2006).

Clearly, there are a number of accounts that can explain structural priming and its associated effects, some perhaps more parsimoniously than that proposed by Chang et al. (2006). We return to our point, however, that none of these accounts also provides clear predictions about how syntactic knowledge is built. Any theory of syntactic development must account for the fact that children operate with abstract syntactic knowledge from a relatively young age, have developed verb‐specific knowledge by this time, and are sensitive to structural priming. In this regard, the dual‐path model remains a strong candidate since both the computational and behavioral evidence supports a theory of structural priming (of which next word prediction is a fundamental feature) that can also, in principle, be used to explain the type of constraints involved in syntax acquisition.

## 5 Where do we go from here? Toward a unified approach to syntax acquisition

In this work, we examined the plausibility of error‐based learning as a mechanism by which children build adult‐like syntactic knowledge, and we concluded that this type of learning—as instantiated in the dual‐path model—can account for both the short‐term phenomenon of structural priming and the long‐term adaptation that results in syntax acquisition. That is not to say, however, that error‐based learning is the only explanation. Worthy of note is the fact that some of the characteristics of the dual‐path model are not unique to error‐based learning but are also properties of statistical learning and implicit learning. For instance, one assumption of the error‐based learning mechanism in the dual‐path model that is also shared by implicit learning is that speakers acquire knowledge from the input without awareness that they are doing so. Another assumption of the model is that speakers use statistics to extract probabilistic information from the input about the frequency with which items co‐occur—the basis of statistical learning. In some respects, we might view error‐based learning as a bridge between the statistical learning and implicit learning literature, in which case an important question for future research concerns how these three strands can inform each other.

For example, an interesting question concerns the role of error‐based learning mechanisms, as opposed to associative learning mechanisms (e.g., Hebbian learning; Hebb, 1949) in both implicit and statistical learning. In error‐based models, it is prediction error, rather than repeated exposure to a particular pattern of activation, that drives learning. But it is not clear how plausible this is as the primary mechanism of syntax learning. At present, opinion is mixed, with some arguing that prediction may have a significant role (e.g., Chang, Kidd, & Rowland, 2013; Johnson, Turk‐Browne, & Goldberg, 2013), and others maintaining that, although prediction might contribute to acquisition, it is not fundamental to this process. For instance, Huettig (2015) claims that children are known to track backwards statistics in speech, and that these backwards transitional probabilities (which cannot be used to make predictions) are more likely to support learning. Mani and Huettig (2012) also argue that word learning can occur without prediction. Their study indicated that 2‐year‐old children's ability at predicting upcoming linguistic input was positively associated with their expressive vocabulary, but that even low producers (whose prediction was poor) were able to understand the sentences in the task. According to Huettig and Guerra (2015), prediction might occur only under certain conditions. In their task, Dutch participants viewed a visual display containing one target noun and three distracter nouns before hearing a sentence that encouraged them to look at the target. Both the amount of time that participants previewed the images and the rate at which the sentence was produced were manipulated. Prediction effects were found in both the normal and slow speech rate conditions when participants had 4 s to preview the images. However, when they had just 1 s to preview the images, the prediction effects were only evident when the speech rate was slow, suggesting that prediction is context‐dependent. Further exploration is, therefore, needed if we want to better understand if and how children use predictive mechanisms like error‐based learning during syntax acquisition.

Fortunately, it seems that the field is already moving in this direction: Lin and Fisher (2017) have recently explored whether prediction as error‐based learning—a potential mechanism for abstract structural priming—can also explain how verb‐structural knowledge is learned. In their study, children and adults received training trials designed to induce double object dative (DOD) structures with some verbs and prepositional object dative (PD) structures with others. They reported that not only did training alter the pre‐existing biases of these verbs for children and adults, but these effects were larger for verb‐structure combinations that were unexpected (e.g., DOD‐biased verb *show* presented in a PD structure). In other words, they found a surprisal effect. That the size of the training effect depended on how likely a verb was to appear in its structure makes it compatible with an approach in which structural priming and verb bias learning are underpinned by a common error‐based learning mechanism. These results go some way towards informing us about the process by which children might link their abstract syntactic knowledge to their knowledge about how verbs behave. Worth bearing in mind, though, is that these results indicate only that surprisal (i.e., large prediction error) can alter verb biases, not whether it affects children's abstract syntactic knowledge. Thus, future studies need to consider the role of surprisal in syntax acquisition.

Current work by Fazekas, Pine, and Rowland (unpublished data) is doing just this using structural priming. Their study involves a pre‐test phase during which children and adults’ verb‐structure preferences are assessed with a set of dative verbs, followed by a priming phase in which they are presented with prime sentences containing verb‐structure mismatches with different verbs (i.e., sentences that should lead to prime surprisal). After the priming phase, a post‐test identical to the pre‐test is run. If it is the case that surprisal (as a consequence of error‐based learning) leads to long‐term changes in syntactic knowledge, then we should see a difference between pre‐ and post‐ test production with the structures produced at post‐test reflecting the structures produced during the priming phase.

For many years now, researchers have approached the process by which children rapidly acquire the abstract syntactic categories of their native language from different viewpoints: in terms of implicit learning, statistical learning and, more recently, prediction and error‐based learning.

While each literature should be recognized in its own right, aligning these associated but separate perspectives is sure to bring about the opportunity for a deeper understanding of the language learning process. Of course, to do so is not straightforward but is, we feel, a step in the right direction towards building a unifying account of syntactic development that considers how children become mature syntax users.
